# Regional citrate versus heparin anticoagulation for continuous renal replacement therapy in critically ill patients: a meta-analysis with trial sequential analysis of randomized controlled trials

**DOI:** 10.1186/s13054-016-1299-0

**Published:** 2016-05-13

**Authors:** Chao Liu, Zhi Mao, Hongjun Kang, Jie Hu, Feihu Zhou

**Affiliations:** Department of Critical Care Medicine, Chinese People’s Liberation Army General Hospital, 28 Fu-Xing Road, Beijing, 100853 People’s Republic of China

**Keywords:** Regional citrate, Heparin, Continuous renal replacement therapy, Anticoagulation, Meta-analysis, Trial sequential analysis

## Abstract

**Background:**

Regional citrate or heparin is often prescribed as an anticoagulant for continuous renal replacement therapy (CRRT). However, their efficacy and safety remain controversial. Therefore, we performed this meta-analysis to compare these two agents and to determine whether the currently available evidence is sufficient and conclusive by using trial sequential analysis (TSA).

**Methods:**

We searched for relevant studies in PubMed, Embase, the Cochrane Library databases and the China National Knowledge Infrastructure (CNKI) Database from database inception until September 2015. We selected randomized controlled trials comparing regional citrate with heparin in adult patients with acute kidney injury (AKI) who were prescribed CRRT.

**Results:**

Fourteen trials (*n* = 1134) met the inclusion criteria. Pooled analyses showed that there was no difference in mortality between the regional citrate and heparin groups (relative risk (RR) 0.97, 95 % confidence interval (CI) 0.84, 1.13, *P* > 0.05), which was confirmed by TSA. Compared with heparin, regional citrate significantly prolonged the circuit life span in the continuous venovenous haemofiltration (CVVH) subgroup (mean difference (MD) 8.18, 95 % CI 3.86, 12.51, *P* < 0.01) and pre-dilution subgroup (MD 17.51, 95 % CI 9.85, 25.17, *P* < 0.01) but not in the continuous venovenous haemodiafiltration (CVVHDF) subgroup (MD 28.60, 95 % CI −3.52, 60.73, *P* > 0.05) or post-dilution subgroup (MD 13.06, 95 % CI −2.36, 28.48, *P* > 0.05). However, the results were not confirmed by TSA. A reduced risk of bleeding was found in the regional citrate compared with the systemic heparin group (RR 0.31, 95 % CI 0.19, 0.51, *P* < 0.01) and TSA provided conclusive evidence. Fewer episodes of heparin-induced thrombocytopoenia (HIT) (RR 0.41, 95 % CI 0.19, 0.87, *P* = 0.02) and a greater number of episodes of hypocalcaemia (RR 3.96, 95 % CI 1.50, 10.43, *P* < 0.01) were found in the regional citrate group. However, TSA did not provide conclusive evidence.

**Conclusion:**

In adult patients with AKI, there is no difference in mortality between the regional citrate and heparin treated groups. However, regional citrate is more efficacious in prolonging circuit life span and reducing the risk of bleeding and should be recommended as the priority anticoagulant for critically ill patients who require CRRT.

**Electronic supplementary material:**

The online version of this article (doi:10.1186/s13054-016-1299-0) contains supplementary material, which is available to authorized users.

## Background

Continuous renal replacement therapy (CRRT) has been widely used in critically ill patients with acute kidney injury (AKI) and anticoagulation of the extracorporeal blood is necessary to maintain the patency of the circuit [1]. In recent decades, different anticoagulation strategies have been used in clinical settings [2] and heparin is the most commonly used anticoagulant. Although heparin has the advantages of low cost, easy monitoring and simple reversal, it may increase bleeding. Additionally, there is the risk of heparin-induced thrombocytopenia type II (HIT-II) that can result in life-threatening complications [3]. Regional citrate anticoagulation (RCA), which was first introduced into clinical use in the early 1980s [4], has been recommended as the most suitable form of CRRT regional circuit anticoagulation [5] and has been safely used even in patients with severe liver dysfunction [6]. However, citrate infusion in critically ill patients impacts a variety of metabolic systems, which can lead to hypocalcaemia, metabolic alkalosis and citrate toxicity. These potential disturbances can be resolved by careful monitoring, adherence to treatment protocols, and oversight by trained staff in clinical practice [7]. Previous meta-analyses [8–10] have evaluated the efficacy and safety of regional citrate versus heparin anticoagulation. However, the results have yielded large discrepancies. Furthermore, a single-centre [11] and two multi-centre [12, 13] randomized controlled trials (RCTs) that were published recently were not included in these meta-analyses. To provide the most recent available evidence, we performed this meta-analysis comparing the two agents. We further applied trial sequential analysis (TSA) to determine whether the currently available evidence was sufficient and conclusive.

## Methods

The Preferred Reporting Items for Systematic Reviews and Meta-Analyses (PRISMA statement) guidelines were used to perform this meta-analysis [14].

### Search strategy and information sources

A search of the PubMed (US National Library of Medicine, Bethesda, MD, USA), Cochrane Library databases, EMBASE and China National Knowledge Infrastructure (www.cnki.net) databases from database inception to September 2015 was performed. Specific search strategies were developed for each database, using different combinations and variations of the search terms “anticoagulation,” “citrate,” “heparin,” “continuous renal replacement therapy (CRRT),” “continuous venovenous haemofiltration (CVVH),” “continuous venovenous hemodialysis (CVVHD),” “continuous venovenous hemodiafiltration (CVVHDF),” and “Randomized Controlled Trial.” The search was limited to human subjects, and no language restrictions were applied. Further searches were performed if necessary by manually reviewing conference proceedings and the references of review articles.

### Inclusion and exclusion criteria

The inclusion criteria were as follows: (1) study design: RCTs; (2) comparison: evaluating the efficacy and safety of regional citrate compared with heparin anticoagulation for CRRT; and (3) population: conducted in critically ill adult patients (>16 years old). Exclusion criteria were as follows: (1) studies including patients with liver failure or hemorrhagic disorders; and (2) data from the published results could not be extracted and analyzed.

### Study selection and data extraction

Two investigators (CL and ZM) independently performed the study selection. Disagreements between the two investigators were resolved by third party adjudication (FZ). A standard form was used to collect data from each study. The form included first author, year of publication, study design, number of patients, number of circuits, patient characteristics, circuit life span and details of complications. The primary outcomes were mortality and circuit life span. Secondary outcomes included bleeding events, HIT, metabolic alkalosis and hypocalcemia.

### Quality assessment

The quality of included studies was assessed by using standard criteria: random sequence generation, allocation concealment, blinding of participants and personnel, blinding of outcome assessment, incomplete outcome data, selective reporting and other bias. When data were missing or incomplete, the original authors were contacted by written correspondence for clarification, and any relevant information obtained was included in the review.

### Grading quality of evidence

Two investigators (CL and ZM) independently assessed the quality of evidence for primary and secondary outcomes according to the Grading of Recommendations Assessment, Development and Evaluation (GRADE) Working Group criteria [15]. Based on risk of bias, indirectness, imprecision, inconsistency and publication bias, the quality of the evidence was classified into four categories (high, moderate, low and very low).

### Statistical analysis

We calculated relative risks (RRs) with 95 % confidence intervals (CIs) for dichotomous outcomes and mean differences (MDs) with 95 % CIs for continuous outcomes. Heterogeneity across studies was quantified using the *I*^*2*^ statistic, and the *I*^*2*^ > 50 % indicated significant heterogeneity [16]. The fixed-effect analytical model was used to pool the results of trials with acceptable or no heterogeneity. The random-effect model was used to analyse the results of trials with significant heterogeneity, and the sensitivity analysis was performed to test the robustness of results. Subgroup analysis was conducted to investigate potential sources of between-study heterogeneity. Publication bias was assessed using the Begg and Egger tests. A *P* value less than 0.05 was considered to indicate a statistically significant difference. All statistical analyses were performed using Review Manager, version 5.1.2 (RevMan, The Cochrane Collaboration, Oxford, UK). If the mean or standard deviation of circuit survival time could not be directly obtained from trials, we extracted the data from published Kaplan-Meier survival curves [17] or estimated the mean and deviation from the sample size, median, range and/or interquartile range [18].

### Trial sequential analysis

In a meta-analysis, random error increases the risk of type I errors when sparse data are analysed and repeated significance testing is conducted for the accumulated data. To minimize this risk, monitoring boundaries were applied to determine if the trial should be terminated early under the condition of an amply small *P* value. This is referred to as TSA [19, 20], a method that combines an a priori information size calculation for a meta-analysis with the adaptation of monitoring boundaries to evaluate the accumulated evidence [21]. When the cumulative *Z*-curve crosses the trial sequential monitoring boundary or enters the futility area, a sufficient level of evidence for the anticipated intervention effect may have been reached, and no further trials are needed. If the *Z*-curve does not cross any of the boundaries and the required information size has not been reached, evidence to reach a conclusion is insufficient, and more trials are needed to confirm the results. We calculated information size as a diversity-adjusted required information size, suggested by the diversity of the intervention effect estimates among the included trials [22]. For our TSA, we estimated the required information size using α = 0.05 (two sided), β = 0.20 (power 80 %), the control event proportions calculated from the heparin group and a relative risk reduction of 20 % in outcomes. If the random-effect model of Sidik-Jonkman (SJ) and DerSimonian-Laird (DL) approaches produced different results, we conducted meta-analyses with the two approaches and considered the implications of each of the two scenarios being true. The software TSA version 0.9 beta (http://www.ctu.dk/tsa) was used for these analyses [23].

## Results

### Study enrolment and characteristics

**Fig. 1:**
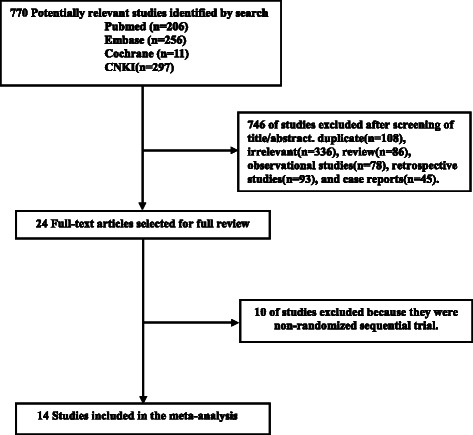
Flow chart of the study selection. *CNKI* Chinese National Knowledge Infrastructure

Seven hundred and seventy potentially relevant studies and 24 articles were retrieved for detailed assessment. Ten articles were excluded because they were non-randomized sequential trials. In total, 14 studies were included in this meta-analysis (Fig. 1).

The characteristics and patient demographic data are summarized in Table 1. All studies consistently included patients with acute renal failure that required CRRT. Patients with severe liver failure, ischaemic hepatitis, high risk of bleeding, severe coagulation disorders, history of heparin allergy and HIT had been excluded from most of the trials. Ten single-centre [11, 24–32] and four multi-centre studies [12, 13, 33, 34] were identified. These trials were reported between 2004 and 2015 and a total of 1134 patients were included in this study. Sample sizes of these trials varied considerably. Only five trials [4, 11–13, 28, 33] included more than 100 patients. Seven trials [12, 24–27, 31, 34] reported the total number of circuits. Baseline characteristics and mean severity scores were similar between the two groups. Nine trials [13, 24, 26–28, 30–33] applied CVVH and four [11, 25, 29, 34] applied CVVHDF. For the control group, ten trials [11, 13, 24, 25, 27, 29, 30, 32–34] used systemic heparin, three [12, 26, 31] regional heparin and one [28] nadroparin. Various citrate protocols and heparin anticoagulation doses were reported in these trials.

**Table 1:** Characteristics of the included trials

Source	Setting	Exclusion	Patients (M/F)/circuits, number	Mean age, years	Severity	Modality; dilution; blood flow (ml/min)	Filter material
Stucker et al. [11] (2015; CH)	ICU of the university hospitals	Cirrhosis, severe coagulopathy, high risk of bleeding and sensitivity to heparin	C: 54 (32/22)/NR	C: 60 ± 14^a^	C: 28 ± 9 (APACHE II)/63 ± 18 (SAPS)^a^	CVVHDF; 2/3 pre-dilution and 1/3 post-dilution; 100–200	1.5 m^2^ High-flux membrane
			H: 49 (32/17)/NR	H: 65 ± 16^a^	H: 29 ± 9 (APACHE II)/65 ± 18 (SAPS)^a^		
Gattas et al. [12] (2015; AU)	Seven different ICUs	Liver failure, pregnant or breastfeeding, HIT, chronic dialysis	C: 105 (74/31)/390	C: 66.4 ± 14.3^a^	C: 25.6 ± 7.6 (APACHE II)^a^	CVVHDF (61 %) CVVH (29 %); pre-dilution; 150 (52 %) 200 (23 %)	Aquarius or Prismaflex
			H: 107 (72/35)/467	H: 66.8 ± 14.9^a^	H: 25.0 ± 6.9 (APACHE II)^a^		
Schilder et al. [13] (2014; NL)	Ten different ICUs	High bleeding risk, other Therapeutic anticoagulation, HIT	C: 66 (44/22)/NR	C: 67 (36–87)^b^	C: 23 (11–53) (APACHE II)/10 (2–19) (SOFA)^b^	CVVH; pre-dilution; 180	NR
			H: 73 (49/24)/NR	H: 67 (23–85)^b^	H: 25 (6–43) (APACHE II)/11 (3–18) (SOFA)^b^		
Brain et al. [25] (2014; AU)	A large metropolitan ICU	Contraindication to citrate or heparin, pregnancy, or lactation	C: 19 (12/7)/96 H: 11 (7/4)/125	C: 64 ± 13^a^ H: 51 ± 17^a^	C: 80 (58–99) (APACHE III)^b^ H: 61 (52.5–91.5) (APACHE III)^b^	CVVHDF; pre-dilution; mean 191 (citrate) and 217 (heparin)	ST-100 (68.8 %); ST-150 (7.2 %); M100 (20.8 %); others (3.2 %)
Monchi et al. [27] (2004; BE)	32-Bed medical and surgical ICU	Cirrhosis, severe coagulopathy, high risk of bleeding	C: 8 (NR)/26	C: 67 (52–77)^b^	C: 40 (31–53) (SAPS)^b^	CVVH; post-dilution; 175	1.6 m^2^ Highly permeable PS membrane
			H: 12 (/NR)/23	H: 64 (52–74)^b^	H: 42 (33–55) (SAPS)^b^		
Lin XM et al. [29] (2007; CN)	Adult mixed ICU	NR	C: 27 (16/11)/NR	C: 63 ± 21^a^	C: 82.5 ± 22.4 (APACHE III)^a^	CVVHDF; pre-dilution; 100–180	PRISMA M-100 AN69
			H: 23 (14/9)/NR	H: 64 ± 19^a^	H: 75.6 ± 18.3 (APACHE III)^a^		
Cui W et al. [30] (2011; CN)	Adult mixed ICU	NR	C: 23 (12/11)/NR	C: 46.9 ± 6.1^a^	C: NR	CVVH; NR; NR	PRISMA
			H: 23 (13/10)/NR	H: 47.2 ± 5.9^a^	H: NR		
Yang ST et al. [31] (2014; CN)	Adult mixed ICU	severe coagulopathy, high risk of bleeding	C: 25 (NR)/81	61.7 ± 8.6	C: NR	CVVH; pre-dilution; 200–250	Aquarius, HF1200
			H: 21 (NR)/53		H: NR		
Oudemans-van Straaten et al. [28] (2009; NL)	ICU of a teaching hospital	Cirrhosis, bleeding, HIT, chronic dialysis, Contraindication to citrate or heparin	C: 97 (66/31)/NR	C: 73 (67–79)^b^	C: 28 (27–30) (APACHE II)/59 (55–62) (SAPS)^b^	CVVH; post-dilution; 220	1.9 m^2^ Cellulose triacetate hollow fibre membrane
			N: 103 (70/33)/NR	N: 73 (67–79)^b^	N: 8 (27–29) (APACHE II)/61 (58–64) (SAPS)^b^		
Betjes et al. [24] (2007; NL)	Adult mixed ICU	HIT, severe coagulopathy, high risk of bleeding, severe circulatory shock and liver failure	C: 1 (15/6)/70	C: 57.8 ± 4.2^a^	C: 51.4 ± 4.1 (SAPS)^a^	CVVH;post-dilution; 150	High-flux triacetate
			H: 27 (19/8)/72	H: 55.2 ± 2.8^a^	H: 51.0 ± 2.6 (SAPS)^a^		
Fealy et al. [26] (2007; AU)	ICU of tertiary hospital	Liver failure, hepatitis and contraindication to citrate or heparin	C:10 (9/1)/10	71 (63.5–76.5)^b^	SAPS: 41 (31–43) APACHE II:17 (15–21)^b^	CVVH; pre-dilution; 150	APS650 PS hollow fibre membrane
			H:10 (9/1)/10				
Kutsogiannis et al. [34] (2005; CA)	Tertiary and community hospital ICU	Liver failure, Contraindication to citrate or heparin	C: 16 (7/9)/36	C: 66.5 ± 14.5^a^	C: 7.75 ± 3.53 (OD)^a^	CVVHDF; pre-dilution; 125	Standard PRISMA M-100 AN69
			H: 14 (8/6)/43	H: 63.9 ± 21.2^a^	H: 9.42 ± 2.31 (OD)^a^		
Tiranathanagul et al. [32] (2011; TH)	Adult mixed ICU	Severe hepatitis and cirrhosis, hypercalcaemia, Contraindication to citrate or heparin, other therapeutic anticoagulation	C: 10 (5/5)/NR	C: 69.5(32–78)^b^	C: 21 (18–29) (APACHE II)^b^	CVVH; pre-dilution; 120	1.5 m^2^ Polyethersulfone dialyzers
			H: 10 (7/3)/NR	H: 75.5 (18–87)^b^	H: 22 (15–29) (APACHE II)^b^		
Hetzel et al. [33] (2011; DE)	Nine different ICUs	Contraindication to citrate or heparin, metabolic, alkalosis, pregnancy or lactation, chronic dialysis, other therapeutic, anticoagulation, HIT	C: 87 (57/30)/NR	C: 62 ± 15.3^a^	C: 21.8 ± 5.1 (APACHE II)	CVVH; pre-dilution; HF-solution flow 3:1	AV600S high-flux membrane
					9.95 ± 2.9 (SOFA)^a^		
			H: 83 (59/24)/NR	H: 65 ± 12.5^a^	H: 22.04 ± 5.5 (APACHE II)		
					9.95 ± 2.6 (SOFA)^a^		

*Abbreviations*: *M* male, *F* female, *APACHE* Acute Physiology and Chronic Health Evaluation II, *AU* Australia, *BE* Belgium, *C* citrate, *CA* Canada, *CH* Switzerland, *CN* China, *CRRT* continuous renal replacement therapy, *CVVH* continuous venovenous haemofiltration, *CVVHDF* continuous venovenous haemodiafiltration, *DE* Germany, *H* heparin, *HF* haemofiltration, *HIT* heparin-induced thrombocytopenia, *N* nadroparin, *NL* the Netherlands, *NR* not reported, *OD* logistic organ dysfunction score, *SAPS* Simplified Acute Physiology II score, *SOFA* Sepsis-Related Organ Failure Assessment score. ^a^Mean ± standard error. ^b^Median (interquartile range)

### Quality of studies

**Fig. 2:**
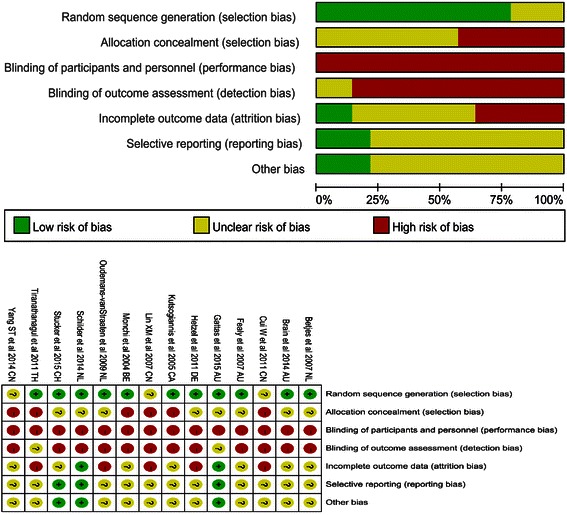
Assessment for risk of bias. *NL* The Netherlands, *AU* Australia, *CN* China, *DE* Germany, *CA* Canada, *BE* Belgium, *CH* Switzerland, *TH* Thailand

The details of the risk of bias tool are shown in Fig. 2. Although all of these studies were RCTs, allocation concealment was not performed. Owing to the nature of the interventions, it was impossible for the medical staff to perform the study blinded. GRADE Working Group grades of evidence were low for primary outcomes and secondary outcomes of adverse events. This was mainly due to risk of bias and small sample sizes within studies. Full GRADE profiles for the included evidence can be found in Additional file 1.

### Primary outcomes

#### Mortality

**Fig. 3:**
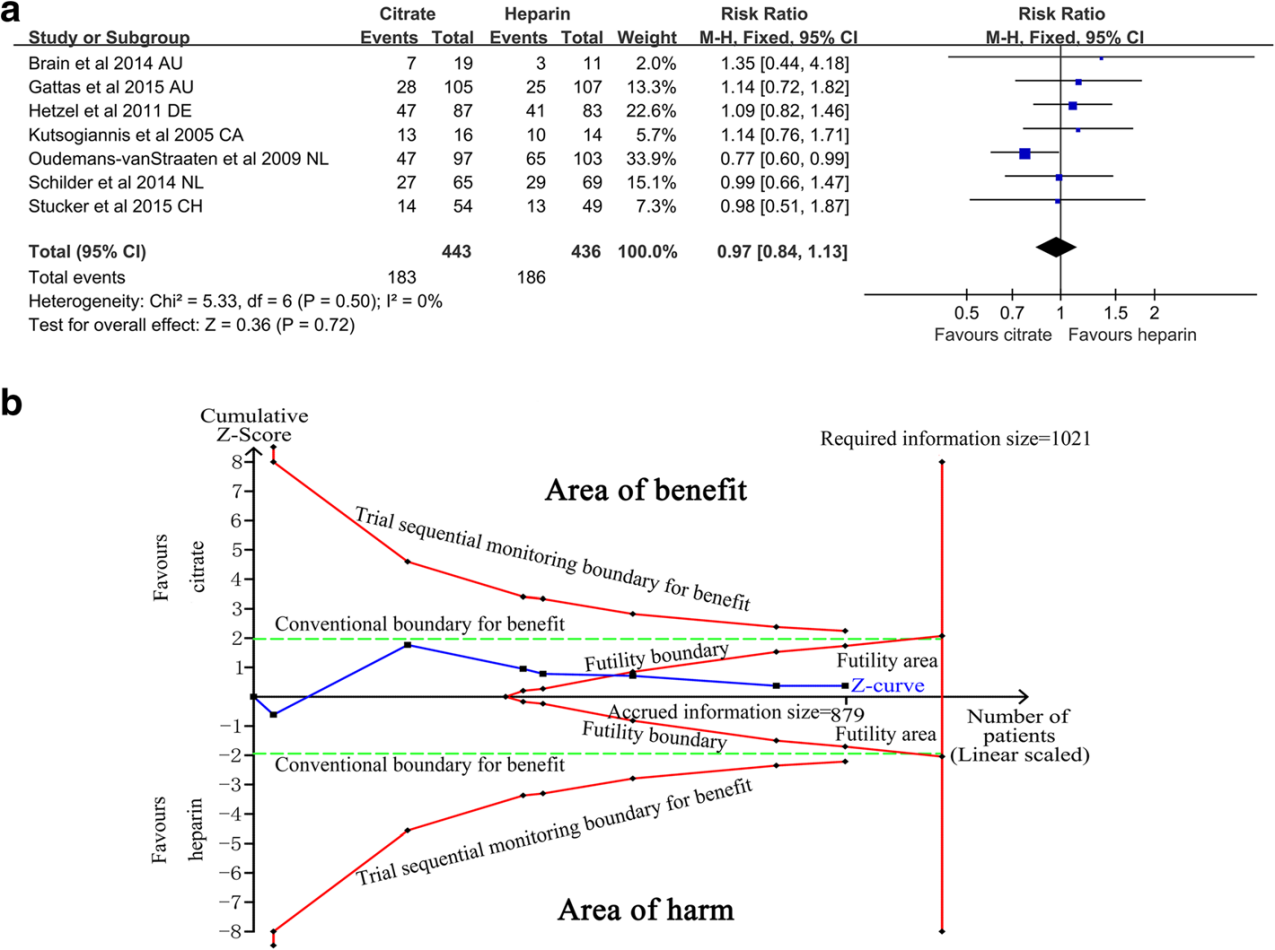
Effect of regional citrate versus heparin anticoagulation on mortality. **a** Mortality. **b** Fixed-effect model of trial sequential analysis for mortality. A diversity-adjusted information size of 1021 participants calculated on the basis of a mortality rate of 42.66 % in the heparin group, relative risk reduction 20 %, α = 5 % (two sided), β = 20 %, *I* ^2^ = 0 %. *Complete blue line* represents cumulative Z-curve, which crossed the futility boundary (*complete red line*) and reached the futility area. *AU* Australia, DE Germany, CA Canada, *NL* The Netherlands, *CH* Switzerland, *M-H* Mantel-Haenszel

The main endpoint of mortality was defined in the individual trials. If mortality was assessed at several time points in a study, we used data from the latest follow-up time for overall mortality assessment. Seven trials [11–13, 25, 28, 33, 34] reported the mortality of patients. Overall mortality in seven trials was 42.0 % (369/879). In the citrate group, 41.3 % (183/443) of patients died compared with 42.7 % (186/436) in the heparin group. There was no significant difference in mortality between the citrate and heparin group (RR 0.97, 95 % CI 0.84, 1.13, *P* = 0.72, Fig. 3a), and no significant heterogeneity was found (Chi^2^ = 5.33, degrees of freedom (df) = 6, *P* = 0.50; *I*^2^ = 0 %) (Fig. 3a). For the low heterogeneity, the fixed-effect model was used for TSA, and the results showed that the cumulative Z-curve crossed the futility boundary and entered the futility area (RR 0.97, 95 % CI 0.84, 1.13, *P* = 0.72) (Fig. 3b), establishing sufficient and conclusive evidence and showing that further trials were not required.

### Circuit life span

**Fig. 4:**
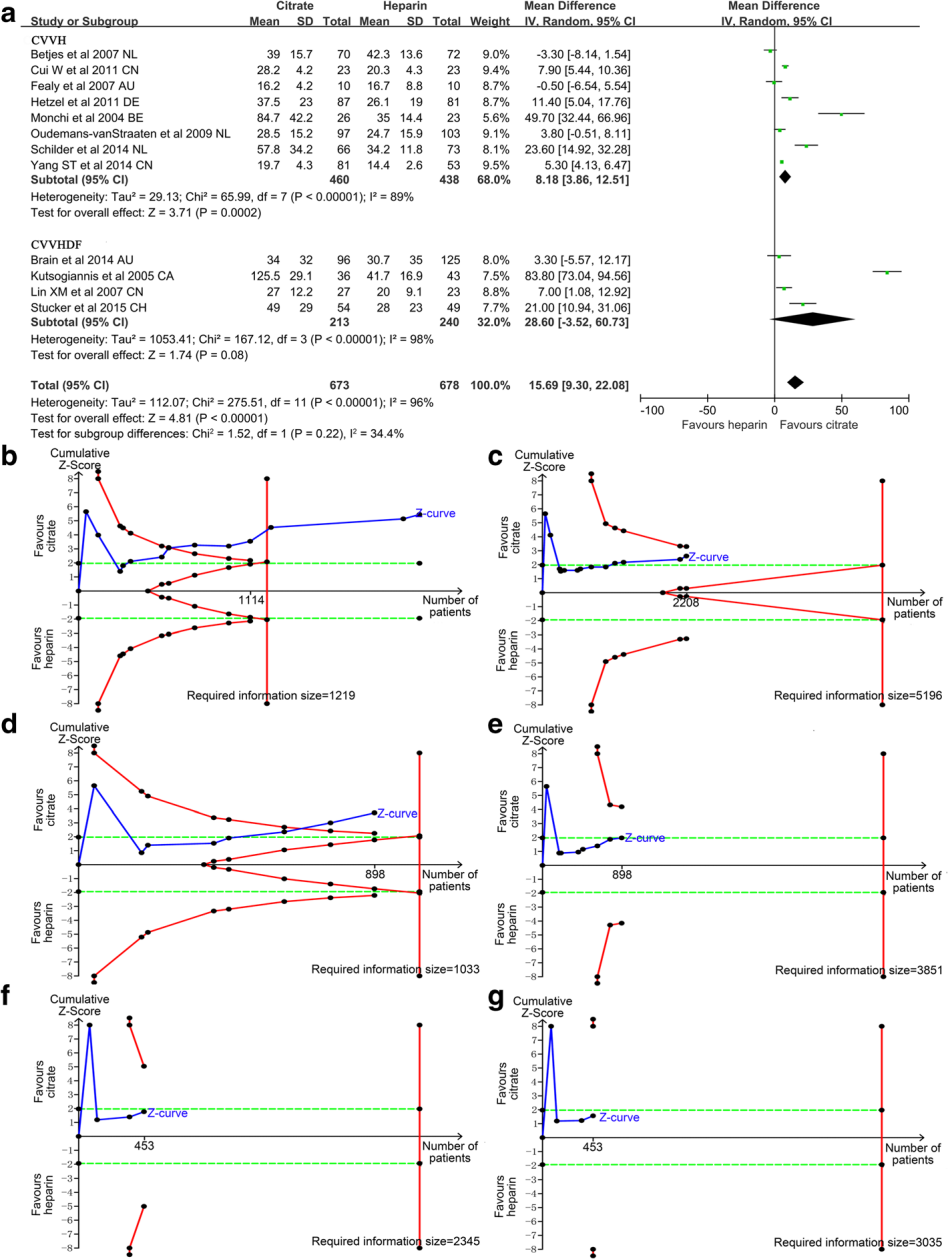
Effect of regional citrate versus heparin anticoagulation on circuit life span (continuous venovenous haemofiltration (CVVH) and continuous venovenous haemodiafiltration (CVVHDF) subgroup analysis). **a** Circuit life span. **b**-**g** *Complete blue line* represents the cumulative Z-curve, *complete red line* represents the trial sequential monitoring boundary for benefit and *etched green line* represents the conventional boundary for benefit. **b** The DerSimonian-Laird (DL) approach used for all trials. A diversity-adjusted information size of 1219 circuits was calculated on the basis of a mean difference (MD) of 15.43, variance of 167.21, *I* ^2^ = 98.11 %, α = 5 % (two sided) and β = 20 %. Cumulative Z-curve crosses the trial sequential monitoring boundary for benefit and reaches the required information size. **c** The Sidik-Jonkman (SJ) approach used for all trials. A diversity-adjusted information size of 5196 circuits was calculated on the basis of a MD of 17.14, variance of 167.21, *I* ^2^ = 99.65 %, α = 5 % (two sided) and β = 20 %. *T*he cumulative Z-curve crosses the conventional boundary for benefit but not the trial sequential monitoring boundary for benefit. **d** The DL approach used for the CVVH subgroup. A diversity-adjusted information size of 1033 circuits was calculated on the basis of a MD of 8.18, variance of 110.0, *I* ^2^ = 94.97 %, α = 5 % (two sided) and β = 20 %. The cumulative Z-curve crosses both the conventional boundary and the trial sequential monitoring boundary. **e** The SJ approach used for the CVVH subgroup. A diversity-adjusted information size of 3851 circuits was calculated on the basis of a MD of 11.08, variance of 110.0, *I* ^2^ = 99.25 %, α = 5 % (two sided) and β = 20 %. The cumulative Z-curve crosses the conventional boundary, but not the trial sequential monitoring boundary. **f**, **g** The DL and SJ approaches used for the CVVHDF subgroup. The cumulative Z-curve does not cross the conventional boundary or the trial sequential monitoring boundary. *NL* The Netherlands, *CN*, China, *AU* Australia, *DE* Germany, *BE* Belgium, *IV *Inverse Variance

**Fig. 5:**
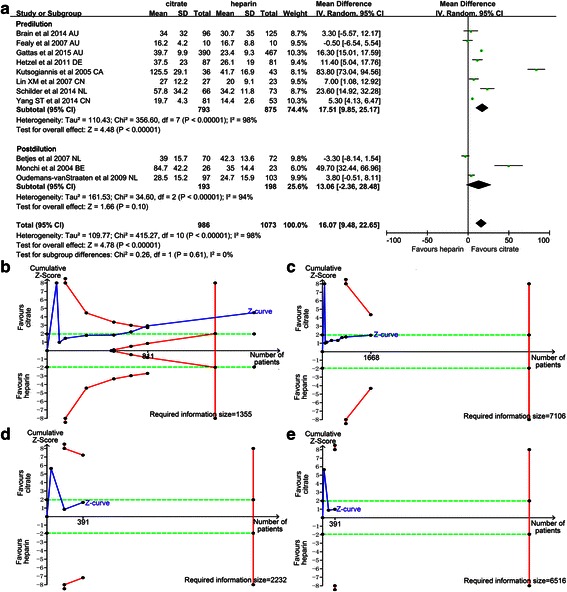
Effect of regional citrate versus heparin anticoagulation on circuit life span (pre-dilution and post-dilution subgroup analysis). **a** Circuit life span. **b**-**e** *Complete blue line* represents the cumulative Z-curve, *complete red line* represents the trial sequential monitoring boundary for benefit and *etched green line* represents the conventional boundary for benefit. **b** The DerSimonian-Laird (DL) approach used for the pre-dilution subgroup (eight trials). A diversity-adjusted information size of 1355 circuits was calculated on the basis of a mean difference (MD) of 17.51, variance of 150.43, *I* ^2^ = 98.82 %, α = 5 % (two sided) and β = 20 %. The cumulative Z-curve crosses the trial sequential monitoring boundary for benefit and reaches the required information size. **c** The Sidik-Jonkman (SJ) approach used for the pre-dilution subgroup (six trials: two trials ignored in the interim looks due to too low information use (<1.0 %)). A diversity-adjusted information size of 7106 circuits was calculated on the basis of a MD of 18.55, variance of 150.43, *I* ^2^ = 99.8 %, α = 5 % (two sided) and β = 20 %. The cumulative Z-curve crosses the conventional boundary but not the trial sequential monitoring boundary. **d** The DL approach used for the post-dilution subgroup (three trials). A diversity-adjusted information size of 2232 circuits was calculated on the basis of a MD of 13.06, variance of 509.29, *I* ^2^ = 95.79 %, α = 5 % (two sided) and β = 20 %. The cumulative Z-curve does not cross the conventional boundary or trial sequential monitoring boundary. **e** The SJ approach used for the post-dilution subgroup (two trials: one trial ignored in the interim looks due to too low information use (<1.0 %)). A diversity-adjusted information size of 6516 circuits calculated on the basis of a MD of 15.76, variance of 509.29, *I* ^2^ = 99.0 %, α = 5 % (two sided) and β = 20 %. The cumulative Z-curve does not cross the conventional boundary or trial sequential monitoring boundary. *AU* Australia, *DE* Germany, *CA*, Canada, *CN*, China, *NL* The Netherlands, *IV* Inverse Variance

Thirteen trials [11–13, 24–31, 33, 34] investigated the circuit life span of the citrate versus heparin groups during CRRT. The circuit life span was significantly longer in the citrate group than in the heparin group, with a mean difference of 15.69 h (95 % CI 9.30, 22.08, *P* < 0.01; *I*^2^ = 96 %, *P* < 0.01) (Fig. 4a). Due to remarkable heterogeneity, two pre-set subgroup analyses were performed for populations with CVVH or CVVHDF and pre-dilution or post-dilution, respectively. Overall, in the CVVH (mean difference (MD) 8.18, 95 % CI 3.86, 12.51, *P* < 0.01; *I*^2^ = 89 %, *P* < 0.01) (Fig. 4a) and pre-dilution (MD 17.51, 95 % CI 9.85, 25.17, *P* < 0.01, *I*^2^ = 98 %, *P* < 0.01) (Fig. 5a) subgroups, the circuit life span was significantly longer in the citrate group than in the heparin group. However, in the CVVHDF (MD 28.60, 95 % CI −3.52, 60.73, *P* = 0.08; *I*^2^ = 98 %, *P* < 0.01 Fig. 4a) and post-dilution (MD 13.06, 95 % CI −2.36, 28.48, *P* = 0.1; *I*^2^ = 94 %, *P* < 0.01 Fig. 5a) subgroups, the circuit life span was similar in the two groups.

For the significant inter-trial heterogeneity, the random-effect model of the DL and SJ methods were used for TSA. When all trials were included, the DL method results showed that the cumulative Z-curve crossed both the conventional boundary for benefit and the trial sequential monitoring boundary for benefit and reached the required information size (Fig. 5b). However, when using the SJ method, three trials [26, 27, 30] were ignored in the interim looks due to too low information use (<1.0 %)%). The results showed that the cumulative Z-curve crossed the conventional boundary for benefit but not the trial sequential monitoring boundary for benefit (Fig. 5c). In the CVVH and pre-dilution subgroups, the DL method results showed that the cumulative Z-curve crossed both the conventional boundary for benefit and the trial sequential monitoring boundary for benefit (Fig. 4d and Fig. 5b). The SJ method results showed the cumulative Z-curve crossed the conventional boundary for benefit but not the trial sequential monitoring boundary for benefit (Fig. 4e and Fig. 5c). In the CVVHDF and post-dilution subgroups, results from the two methods showed that the cumulative Z-curve did not cross the conventional boundary for benefit and did not enter the futility boundary.

### Secondary outcomes

#### Adverse events

The adverse events included bleeding events, HIT, metabolic alkalosis and hypocalcaemia (Table 2). For acceptable heterogeneity, the fixed-effect analytical model was used to pool the results. Compared with systemic heparin, regional citrate was more efficacious in decreasing the risk of bleeding, which was confirmed by TSA (the cumulative Z-curve crossed both the conventional boundary for benefit and the trial sequential monitoring boundary for benefit (see Additional file 2)). However, there was no significant difference between the regional citrate and regional heparin groups.

**Table 2:** Direct comparison of regional citrate with heparin on adverse events

Adverse events	No. of studies	No. of patients		RR(95%CI)	Heterogeneity	Test for effect (p value)
		Citrate	Heparin		I^2^ (p value)	
Bleeding events	10 (11, 13, 24, 25, 27, 28, 29, 32, 33, 34)^a^	405	405	0.31(0.19, 0.51)	0% (0.56)	<0.00001
	3 (12, 26, 31)^b^	140	138	0.23 (0.03, 1.97)	0% (0.75)	0.18
HIT	5 (11, 12, 13, 28, 33)	409	415	0.41 (0.19, 0.87)	0% (0.73)	0.02
Metabolic alkalosis	7(11, 13, 24, 27, 28, 29, 34)	289	301	0.84 (0.47, 1.49)	40% (0.14)	0.55
Hypocalcemia	7 (11, 24, 27, 28, 29, 33, 34)	310	311	3.96 (1.50, 10.43)	0% (1.00)	0.005

*CI* confidence interval, *HIT* heparin induced thrombocytopenia, *RR* relative risk, ^a^ citrate versus systemic heparin; ^b^ citrate versus regional heparin

Although more HIT events were found in the heparin group, the difference was not confirmed by TSA (see Additional file 2).

The risk of metabolic alkalosis was similar between these two groups, although TSA could not be performed due to too few data. More episodes of hypocalcaemia were reported in the citrate group. Again, TSA also could not be performed due to too few data.

### Cost-effectiveness

Two trials [13, 26] analysed the cost of each treatment. Fealy et al. [26] reported that regional citrate might yield a somewhat longer circuit life. However, the magnitude of the gain in circuit life did not appear to be sufficient to offset the additional cost associated with the use of citrate. Schilder et al. [13] noted that the costs of the first 72 hours of prescribed CVVH were lower in citrate-based CVVH, which could be attributed to the lower cost of filter sets and less labour due to the use of fewer filters during treatment with citrate.

### Inflammatory cytokines

Gattas et al. [12] reported that there was no significant difference between the citrate and heparin groups in the change of circulating levels of interleukin (IL)-6, IL-8 and IL-10 between randomization and the period 48–72 hours later. Tiranathanagul et al. [32] compared the change in myeloperoxidase (MPO) and cytokine production in patients with AKI undergoing CVVH treatment. This RCT enrolled 20 patients who were randomized into a regional citrate group (*n* = 10) and a heparin group (*n* = 10). The results showed that serum MPO and IL-8 levels were significantly decreased in the citrate group. However, there was no survival benefit identified.

### Publication bias

We assessed the potential publication bias for the primary outcomes of mortality (*P* = 1.000 for the Begg test, *P* = 0.209 for the Egger test) and circuit life span (*P* = 0.150 for the Begg test, *P* = 0.361 for the Egger test). No potential publication bias was observed among the included trials (see Additional file 3). Tests were not available for all subgroup datasets for small sample sizes.

## Discussion

This updated meta-analysis with the largest sample size to date found: (1) there was no significant difference in mortality between the two groups, which was confirmed by TSA; (2) RCA significantly prolonged the circuit life span in both the CVVH subgroup and pre-dilution subgroup, although the TSA did not confirm this result; (3) compared with systemic heparin, RCA significantly decreased the bleeding risk, and the result was confirmed by TSA; (4) the incidence of metabolic alkalosis was similar in these two groups; (5) although more episodes of hypocalcaemia were observed in the citrate group, no significant hypocalcaemia-related adverse events were reported and (6) the cost was not significantly increased in the citrate group.

Previous meta-analyses evaluating this topic have been published. However, there are a number of differences between the present study and the previously published meta-analyses. First, this meta-analysis included an additional eight trials performed since 2011. Furthermore, Chinese trials were also included. One systematic review [35] suggested that to include more evidence in meta-analyses, clinical research published by scientists who write in their native language rather than in English, must be taken into account. Thus, the present meta-analysis represents the latest and most comprehensive study. Second, TSA was used to provide more conservative estimates and to better establish sufficient and conclusive evidence. Third, we evaluated the quality of evidence for outcomes based on GRADE Working Group criteria. The body of evidence will aid physicians in making clinical decisions.

In this meta-analysis, mortality was not significantly different between the two types of anticoagulants, and the TSA results suggested that further trials were not required. Improving mortality is the ultimate goal of developing new adjuvant therapy. However, anticoagulation with regional citrate or heparin did not impact the survival rate. Thus, other methods that may decrease mortality should be investigated.

This meta-analysis suggested that RCA may have an advantage in prolonging the circuit life span, especially in the CVVH and pre-dilution subgroups. The circuit life span is influenced by many factors, such as the patient’s clinical condition, coagulation status, the position and patency of the vascular access, the choice of anticoagulant, modality of CRRT and filtration fraction [36]. These factors may also cause heterogeneity among trials. For the significant inter-trial heterogeneity, the SJ and DL methods were used to conduct TSA, with the former being more reliable. TSA results suggested that additional well-designed clinical trials are needed. Wu et al. [37] reported that RCA plus low-dose dalteparin (40.4 ± 30.9 h) prolonged filter run time compared with RCA (21.2 ± 13.5 h, *P* = 0.006) only or normal-dose dalteparin (25.1 ± 24.0 h, *P* = 0.040) only, without increasing the incidence of anticoagulation-related complications. This may, therefore, represent a new anticoagulant approach to use in patients undergoing CRRT.

Four main adverse events were reported in this study. Although this meta-analysis excluded patients with liver failure, several studies have reported that RCA can be safely used in patients with liver failure and patients who are at a high risk of bleeding during CRRT [38, 39]. One observational study [38], which evaluated the safety and efficacy of RCA in ICU patients with liver failure, concluded that RCA-CVVHD can be safely used in patients with liver failure. Furthermore, the authors suggested that RCA can be recommended as first-line anticoagulation for the majority of ICU patients. In addition, Shaikh et al. [39] reported that CVVH with citrate-containing replacement solution is safe and efficacious for critically ill patients with AKI, who are at high risk of bleeding. In terms of the mechanism of anticoagulation, citrate acts as an anticoagulant in the extracorporeal system through chelation of ionized calcium. When the ionized calcium level is less than 0.35 mmol/L, the coagulation process will be interrupted. When ionized calcium enters the systemic circulation; one molecule of citrate will be metabolized into three molecules of bicarbonate by the liver, muscle and kidney and will affected the acid–base status, thus increasing the risk of hypocalcaemia [40]. Although this meta-analysis showed that more episodes of hypocalcaemia were found in the citrate group, no significant hypocalcaemia-related adverse events were reported. Furthermore, the ionized calcium level was easily identified and controlled with monitoring.

The narrative result of cost-effectiveness in our meta-analysis showed that CRRT with RCA had a time-saving effect and helped to decrease the workload. The findings of one observational study [41] are consisted with our result. The authors performed a cost-effectiveness analysis comparing citrate and heparin treatment and found lower haemofilter-related costs and fewer anticoagulation-associated complications, which minimized the total CVVHDF cost (heparin, US$1,209 ± 517/day; citrate, US$757 ± 268/day; *P* < 0.01). Cost-effectiveness is a critical issue when choosing anticoagulants during CRRT for critically ill patients with AKI. However, only two RCTs evaluated cost-effectiveness differences between the two groups. Future studies should therefore pay more attention to the issue of cost-effectiveness.

Our meta-analysis showed that the changes in IL-6, IL-8 and IL-10 were not significantly different between the two groups [12]. Another study [42] reported that the plasma level of neutrophil gelatinase-associated lipocalin (NGAL) in critically ill patients with AKI was not affected by CVVH or the anticoagulation employed. Schilder et al. [43] confirmed that, compared to the heparin group, less C5a and endothelial MPO were released in the regional citrate group. Inflammation and oxidative stress play important roles in the initiation and extension phases of AKI [44]. Therefore, regional anticoagulation with citrate may decrease the inflammatory response during CVVH in critically ill patients with AKI, and may have some benefit for patient survival.

Our analysis has some limitations. First, various modalities of CRRT and RCA protocols were used and caused large clinical heterogeneity among these trials. According to the clinical characteristics, we therefore performed subgroup analyses to reduce and interpret clinical heterogeneity. Second, double-blinding was not performed because of the features of the trials, which may result in performance and detection bias. Thus, we used the GRADE approach to provide objective levels of the body of evidence.

## Conclusion

Between the regional citrate and heparin groups, no significance difference was found in mortality, hypocalcaemia–related adverse events or inflammatory clearance. However, regional citrate is more efficacious in prolonging circuit life span and reducing the risk of bleeding. Therefore, citrate should be recommended as the priority anticoagulant for critically ill patients who require CRRT.

## Key messages

- Patient mortality was similar for regional citrate and heparin anticoagulation during CRRT in the critically ill patient with AKI, which was confirmed by TSA
- RCA significantly prolonged the circuit life span in both the CVVH subgroup and pre-dilution subgroup, although the TSA did not confirm this result
- Compared with systemic heparin, RCA significantly decreased the bleeding risk, and the result was confirmed by TSA

## Additional files

Additional file 1: Clinical evidence profiles (GRADE). (DOCX 19 kb) Additional file 2: TSA for bleeding events and HIT. **A** Fixed-effect model of trial sequential analysis for bleeding events (regional citrate versus systemic heparin). A diversity-adjusted information size of 4061 participants calculated on the basis of a risk of 15.06 % in the heparin group, relative risk reduction (RRR) of 20 %, α = 5 % (two sided), β = 20 % and *I* ^2^ = 0 %. *Complete blue line* represents cumulative Z-curve, which crossed both the conventional boundary (*etched green line*) and the trial sequential monitoring boundary (*complete red line*). **B** Fixed-effect model of trial sequential analysis for HIT events. A diversity-adjusted information size of 13381 participants calculated on the basis of a risk of 5.06 % in the heparin group, RRR of 20 %, α = 5 % (two sided), β = 20 % and *I* ^2^ = 0 %. *Complete blue line* represents cumulative Z-curve, which crossed the conventional boundary (*etched green line*) but not the sequential monitoring boundary (*complete red line*). (PDF 198 kb) Additional file 3: Publication bias for the primary outcomes. **A** Begg's funnel plot for mortality. **b** Begg's funnel plot for circuit life span. (PDF 144 kb)

## Abbreviations

AKI: acute kidney injury
AU: Australia
BE: Belgium
CA: Canada
CH: Switzerland
CI: confidence interval
CN: China
CRRT: continuous renal replacement therapy
CVVH: continuous venovenous haemofiltration
CVVHDF: continuous venovenous haemodiafiltration
DE: Germany
DL: DerSimonian-Laird
GRADE: Grading of Recommendations Assessment, Development, and Evaluation
HIT: heparin-induced thrombocytopenia
IL: interleukin
MPO: myeloperoxidase
MD: mean difference
NL: The Netherlands
PRISMA: Preferred Reporting Items for Systematic Reviews and Meta-Analyses
RCA: Regional citrate anticoagulation
RCT: randomized controlled trial
RR: relative risk
SJ: Sidik-Jonkman
TSA: trial sequential analysis
