# Supplementary material for: Regional citrate versus heparin anticoagulation for continuous renal replacement therapy in critically ill patients: a meta-analysis with trial sequential analysis of randomized controlled trials
Source: Crit Care. 2016 May 13;20:144. doi: 10.1186/s13054-016-1299-0 (PMC4866420; doi:10.1186/s13054-016-1299-0)
Supplement: Additional file 1: — Clinical evidence profiles (GRADE). (DOCX 19 kb) [file 13054_2016_1299_MOESM1_ESM.docx]

**Clinical Evidence Profiles (****GRADE)**

| **Table 1: Regional citrate vs heparin anticoagulation for CRRT in critically ill patients** | | | | | | | | | | | |
| --- | --- | --- | --- | --- | --- | --- | --- | --- | --- | --- | --- |
| **Quality assessment** | | | | | | | **Summary of Findings** | | | | |
| **Participants (studies) Follow up** | **Risk of bias** | **Inconsistency** | **Indirectness** | **Imprecision** | **Publication bias** | **Overall quality of evidence** | **Study event rates (%)** | | **Relative effect** (95% CI) | **Anticipated absolute effects** | |
|  |  |  |  |  |  |  | **With Heparin** | **With Regional citrate** |  | **Risk with Heparin** | **Risk difference with Regional citrate** (95% CI) |
| **Mortality** | | | | | | | | | | | |
| 879 (7 studies) | serious^1^ | no serious inconsistency | no serious indirectness | serious^2^ | undetected | ⊕⊕⊝⊝ **LOW**^1,2^ due to risk of bias, imprecision | 186/436  (42.7%) | 183/443  (41.3%) | **RR 0.97**  (0.84 to 1.13) | **427 per 1000** | **13 fewer per 1000** (from 68 fewer to 55 more) |
| **Circuit life span** (Better indicated by lower values) | | | | | | | | | | | |
| 1351 (12 studies) | serious^1^ | serious^3^ | no serious indirectness | no serious imprecision | undetected | ⊕⊕⊝⊝ **LOW**^1,3^ due to risk of bias, inconsistency | 678 | 673 | **-** |  | The mean circuit life span in the intervention groups was **15.69 higher** (9.3 to 22.08 higher) |
| **Circuit life span - CVVH** (Better indicated by lower values) | | | | | | | | | | | |
| 898 (8 studies) | serious^1^ | serious^3^ | no serious indirectness | no serious imprecision | undetected | ⊕⊕⊝⊝ **LOW**^1,3^ due to risk of bias, inconsistency | 438 | 460 | **-** |  | The mean circuit life span - cvvh in the intervention groups was **8.18 higher** (3.86 to 12.51 higher) |
| **Circuit life span - CVVHDF** (Better indicated by lower values) | | | | | | | | | | | |
| 453 (4 studies) | serious^1^ | serious^3^ | no serious indirectness | serious^2^ | undetected | ⊕⊝⊝⊝ **VERY LOW**^1,2,3^ due to risk of bias, inconsistency, imprecision | 240 | 213 | **-** |  | The mean circuit life span - cvvhdf in the intervention groups was **28.6 higher** (3.52 lower to 60.73 higher) |
| **Bleeding events** | | | | | | | | | | | |
| 1088 (13 studies) | serious^1^ | no serious inconsistency | no serious indirectness | serious^2^ | undetected | ⊕⊕⊝⊝ **LOW**^1,2^ due to risk of bias, imprecision | 64/543  (11.8%) | 17/545  (3.1%) | **RR 0.3**  (0.19 to 0.49) | **Study population** | |
|  |  |  |  |  |  |  |  |  |  | **118 per 1000** | **83 fewer per 1000** (from 60 fewer to 95 fewer) |
|  |  |  |  |  |  |  |  |  |  | **Moderate** | |
|  |  |  |  |  |  |  |  |  |  | **83 per 1000** | **58 fewer per 1000** (from 42 fewer to 67 fewer) |
| **Bleeding events - citrate versus systemic heparin** | | | | | | | | | | | |
| 810 (10 studies) | serious^1^ | no serious inconsistency | no serious indirectness | serious^2^ | undetected | ⊕⊕⊝⊝ **LOW**^1,2^ due to risk of bias, imprecision | 61/405  (15.1%) | 17/405  (4.2%) | **RR 0.31**  (0.19 to 0.51) | **Study population** | |
|  |  |  |  |  |  |  |  |  |  | **151 per 1000** | **104 fewer per 1000** (from 74 fewer to 122 fewer) |
|  |  |  |  |  |  |  |  |  |  | **Moderate** | |
|  |  |  |  |  |  |  |  |  |  | **110 per 1000** | **76 fewer per 1000** (from 54 fewer to 89 fewer) |
| **Bleeding events - citrate versus regional heparin** | | | | | | | | | | | |
| 278 (3 studies) | serious^1^ | no serious inconsistency | no serious indirectness | serious^2^ | undetected | ⊕⊕⊝⊝ **LOW**^1,2^ due to risk of bias, imprecision | 3/138  (2.2%) | 0/140  (0%) | **RR 0.23**  (0.03 to 1.97) | **Study population** | |
|  |  |  |  |  |  |  |  |  |  | **22 per 1000** | **17 fewer per 1000** (from 21 fewer to 21 more) |
|  |  |  |  |  |  |  |  |  |  | **Moderate** | |
|  |  |  |  |  |  |  |  |  |  | **9 per 1000** | **7 fewer per 1000** (from 9 fewer to 9 more) |
| **heparin induced thrombocytopenia(HIT)** | | | | | | | | | | | |
| 824 (5 studies) | serious^1^ | no serious inconsistency | no serious indirectness | serious^2^ | undetected | ⊕⊕⊝⊝ **LOW**^1,2^ due to risk of bias, imprecision | 21/415  (5.1%) | 8/409  (2%) | **RR 0.41**  (0.19 to 0.87) | **Study population** | |
|  |  |  |  |  |  |  |  |  |  | **51 per 1000** | **30 fewer per 1000** (from 7 fewer to 41 fewer) |
|  |  |  |  |  |  |  |  |  |  | **Moderate** | |
|  |  |  |  |  |  |  |  |  |  | **41 per 1000** | **24 fewer per 1000** (from 5 fewer to 33 fewer) |
| **Metabolic alkalosis** | | | | | | | | | | | |
| 590 (7 studies) | serious^1^ | no serious inconsistency | no serious indirectness | serious^2^ | undetected | ⊕⊕⊝⊝ **LOW**^1,2^ due to risk of bias, imprecision | 22/301  (7.3%) | 17/289  (5.9%) | **RR 0.84**  (0.47 to 1.49) | **73 per 1000** | **12 fewer per 1000** (from 39 fewer to 36 more) |
| **Hypocalcemia** | | | | | | | | | | | |
| 621 (7 studies) | serious^1^ | no serious inconsistency | no serious indirectness | serious^2^ | undetected | ⊕⊕⊝⊝ **LOW**^1,2^ due to risk of bias, imprecision | 3/311  (0.96%) | 17/310  (5.5%) | **RR 3.96**  (1.5 to 10.43) | **Study population** | |
|  |  |  |  |  |  |  |  |  |  | **10 per 1000** | **29 more per 1000** (from 5 more to 91 more) |
|  |  |  |  |  |  |  |  |  |  | **Moderate** | |
|  |  |  |  |  |  |  |  |  |  | **0 per 1000** | **-** |

^1^ Owing to the nature of the interventions, it was impossible for the medical staff to perform the study blinded; ^2^ not reached the estimation of sample size; ^3^ I2>50%.

Quality of Evidence High: RCT(s) with no limitations, consistent, precise, and directly applicable results without evidence of reporting bias; Moderate (downgraded from high): RCT(s) with important limitations; Low (downgraded two levels from high): RCT(s) with very serious limitations; Very low (downgraded three levels from high): RCT(s) with very serious limitations and inconsistent results.
