# Supplementary material for: Regional citrate versus heparin anticoagulation for continuous renal replacement therapy in critically ill patients: a meta-analysis with trial sequential analysis of randomized controlled trials
Source: Crit Care. 2016 May 13;20:144. doi: 10.1186/s13054-016-1299-0 (PMC4866420; doi:10.1186/s13054-016-1299-0)
Supplement: Additional file 2: — TSA for bleeding events and HIT. A Fixed-effect model of trial sequential analysis for bleeding events (regional citrate versus systemic heparin). A diversity-adjusted information size of 4061 participants calculated on the basis of a risk of 15.06 % in the heparin group, relative risk reduction (RRR) of 20 %, α = 5 % (two sided), β = 20 % and I 2 = 0 %. Complete blue line represents cumulative Z-curve, which crossed both the conventional boundary (etched green line) and the trial sequential monitoring boundary (complete red line). B Fixed-effect model of trial sequential analysis for HIT events. A diversity-adjusted information size of 13381 participants calculated on the basis of a risk of 5.06 % in the heparin group, RRR of 20 %, α = 5 % (two sided), β = 20 % and I 2 = 0 %. Complete blue line represents cumulative Z-curve, which crossed the conventional boundary (etched green line) but not the sequential monitoring boundary (complete red line). (PDF 198 kb) [file 13054_2016_1299_MOESM2_ESM.pdf]

TSA for bleeding events and HIT.

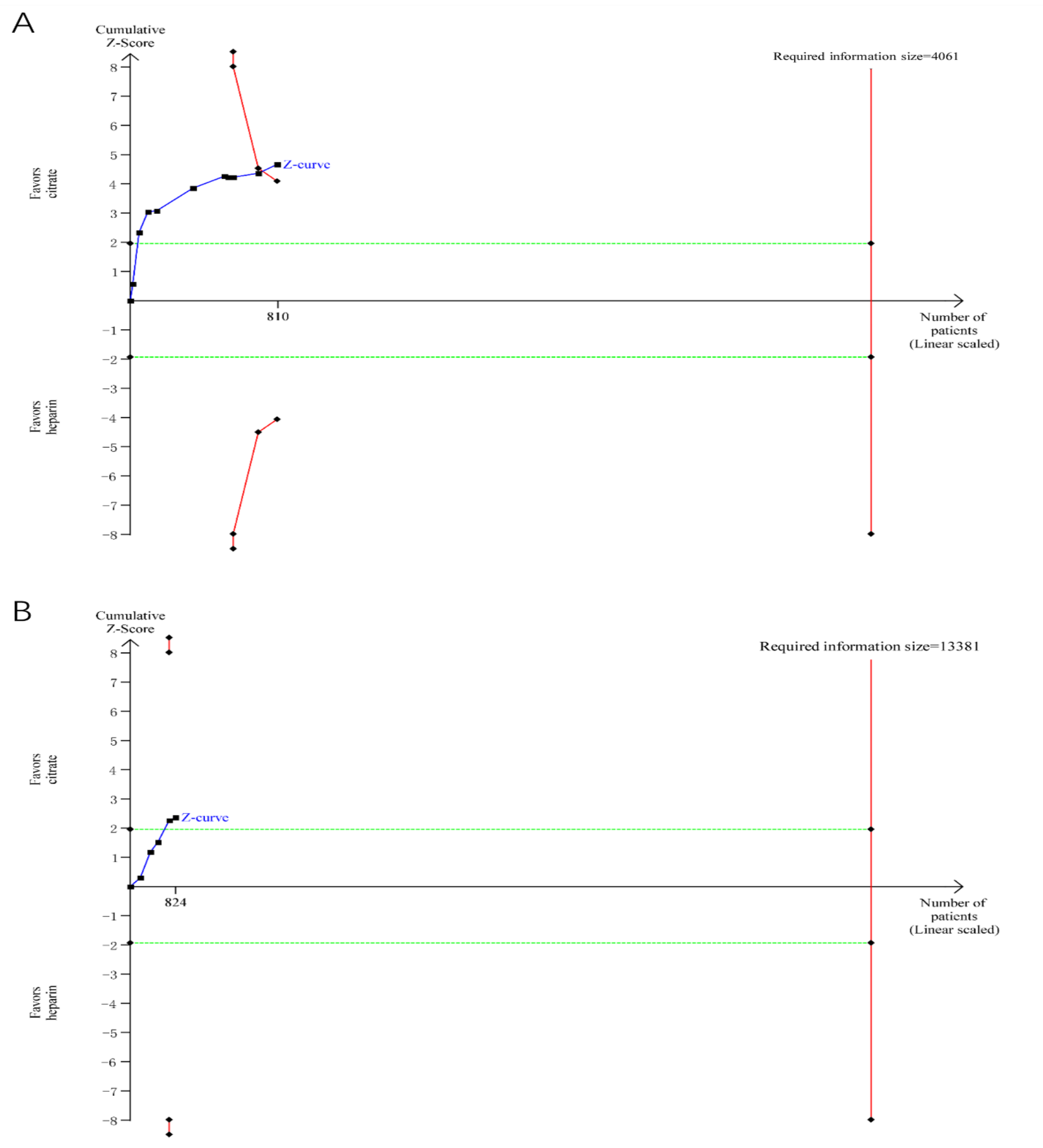

A: Fixed-effect model of trial sequential analysis for bleeding events (regional citrate versus systemic heparin). A diversity adjusted information size of 4061 participants calculated on the basis of a risk of 15.06% in the heparin group, relative risk reduction (RRR) of 20%, α=5% (two sided), β=20%, and I^2=0%. Full blue cumulative Z-curve crossed both the etched green conventional boundary and the full red trial sequential monitoring boundary. **B:** Fixed-effect model of trial sequential analysis for HIT events. A diversity adjusted information size of 13381 participants calculated on the basis of a risk of 5.06% in the heparin group, RRR of 20%, α=5% (two sided), β=20%, and I^2=0%. Full blue cumulative Z-curve crossed the etched green conventional boundary but not the full red trial sequential monitoring boundary.
