# Supplementary material for: Regional citrate versus heparin anticoagulation for continuous renal replacement therapy in critically ill patients: a meta-analysis with trial sequential analysis of randomized controlled trials
Source: Crit Care. 2016 May 13;20:144. doi: 10.1186/s13054-016-1299-0 (PMC4866420; doi:10.1186/s13054-016-1299-0)
Supplement: Additional file 3: — Publication bias for the primary outcomes. A Begg's funnel plot for mortality. b Begg's funnel plot for circuit life span. (PDF 144 kb) [file 13054_2016_1299_MOESM3_ESM.pdf]

Publication bias for the primary outcomes

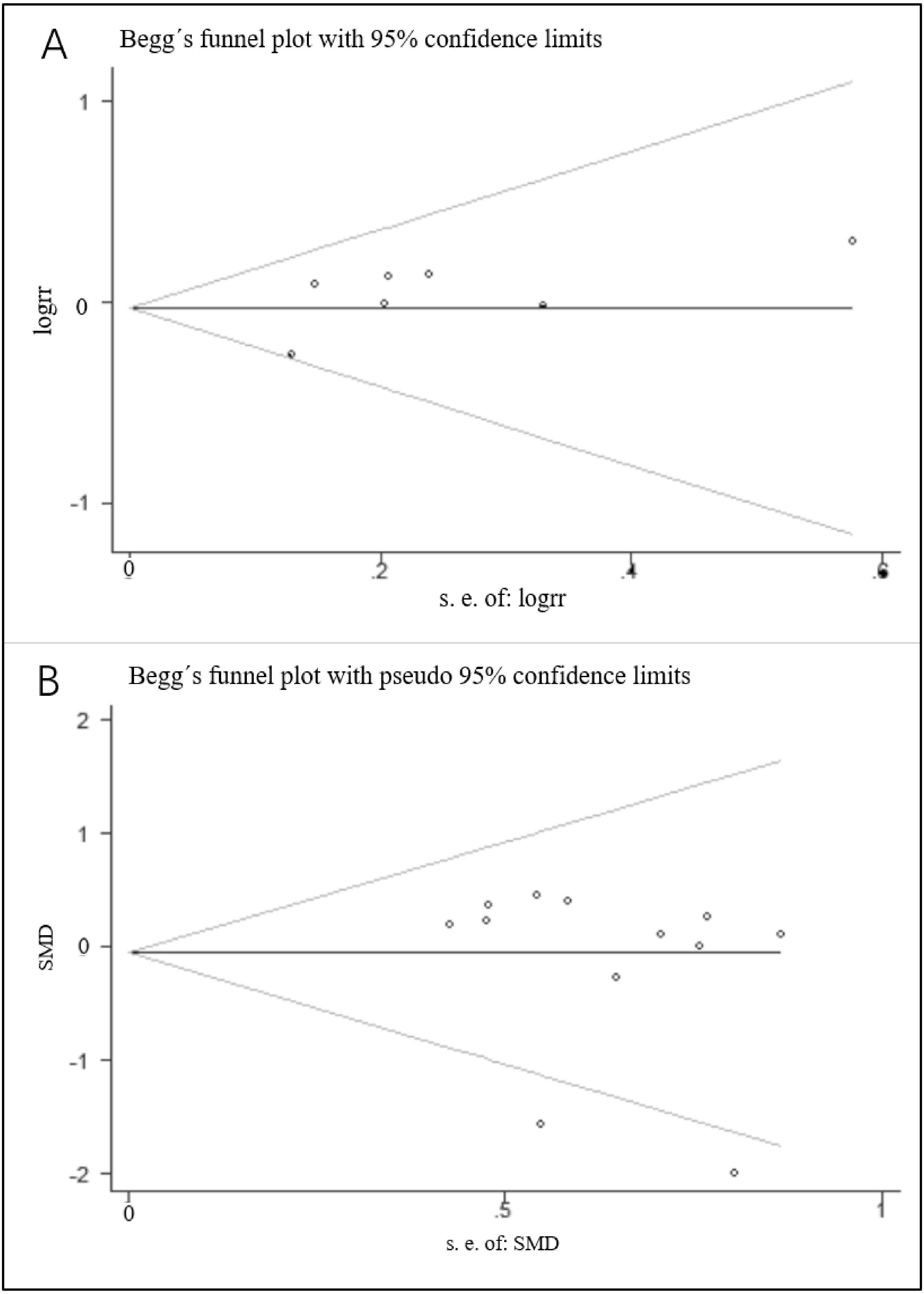

A: Beeg's funnel plot for mortality; **B:** Beeg's funnel plot for circuit life span.
